# Nematicidal Activity of Essential Oil from Lavandin (*Lavandula* × *intermedia* Emeric ex Loisel.) as Related to Chemical Profile

**DOI:** 10.3390/molecules26216448

**Published:** 2021-10-26

**Authors:** Trifone D’Addabbo, Sebastiano Laquale, Maria Pia Argentieri, Maria Grazia Bellardi, Pinarosa Avato

**Affiliations:** 1 Institute for Sustainable Plant Protection, National Council of Research, 70125 Bari, Italy; 2 School of Agricultural, Forest, Food and Environmental Sciences, University of Basilicata, 85100 Potenza, Italy; sebastianolaqual@gmail.com; 3 Department of Pharmacy-Drug Sciences, University of Bari Aldo Moro, 70125 Bari, Italy; mariapia.argentieri@uniba.it (M.P.A.); pinarosa.avato@uniba.it (P.A.); 4 Department of Agricultural Sciences, University of Bologna, 40127 Bologna, Italy; mariagrazia.bellardi@unibo.it

**Keywords:** essential oils, *Lavandula hybrida*, nematicidal activity, *Meloidogyne incognita*, *Pratylenchus vulnus*, sustainable management

## Abstract

Essential oils (EOs) from lavandin are known for a large spectrum of biological properties but poorly and contrastingly documented for their activity against phytoparasitic nematodes. This study investigated the toxicity of EOs from three different lavandin cultivars, Abrialis, Rinaldi Cerioni, and Sumiens, either to juveniles (*J2*) and eggs of the root-knot nematode *Meloidogyne incognita* and to infective stages of the lesion nematode *Pratylenchus vulnus*. The suppressive activity of treatments with EOs from the three lavandin cultivars in soil infested by *M. incognita* was also investigated in a greenhouse experiment on potted tomato. The compositional profiles of tested EOs were also analyzed by GC-FID and GC-MS. Linalool was the major component of all the three EOs, as accounting for about 66%, 48%, and 40% of total EO from *cv* Rinaldi Cerioni, Sumiens, and Abrialis, respectively. Linalool acetate was the second most abundant compound in the EOs from *cv* Abrialis (18.3%) and Sumiens (14.9%), while significant amounts of camphor (11.5%) and 1,8-cineole (12.1%) were detected in *cv* Rinaldi Cerioni and Sumiens EOs, respectively. The mortality of *M. incognita J2* peaked 82.0%, 95.8%, and 89.8% after a 24 h treatment with 100 mg·mL^−1^ solutions of *cv* Abrialis, Rinaldi Cerioni, and Sumiens EOs, respectively. Infective specimens of *P. vulnus* were largely more sensitive than *M. incognita J2*, as there were peak mortality rates of 65.5%, 67.7%, and 75.7% after 4 h of exposure to Abrialis, Rinaldi Cerioni, and Sumiens EO, respectively. All three lavandin EOs significantly affected also *M. incognita* egg hatchability, which reduced to 43.6% after a 48 h egg mass exposure to a 100 µg·mL^−1^ solution of *cv* Rinaldi Cerioni EO. Soil treatments with the three lavandin EOs strongly reduced, according to a dose–effect relationship, density of *M. incognita* eggs, and *J2* both on tomato roots and in soil, as well as significantly reduced gall formation on tomato roots. Finally, almost all soil treatments with the lavandin EOs also resulted in a positive impact on tomato plant growth.

## 1. Introduction

Phytoparasitic nematodes are estimated to cause about 12–15% of world annual crop losses, although their microscopical size and the non-specific symptoms of their presence frequently lead to an underestimation of their dangerousness [1]. The need of environment-safer alternatives to synthetic products traditionally used for phytonematode management has led to increasing evaluations of the potential use of plant-derived biocidal products, which include essential oils (EOs) [2,3,4].

The investigation of nematicidal properties of EOs has been a popular research topic throughout the last decades, and the activity of a huge number of EOs against phytoparasitic nematodes has been extensively documented [2,5,6,7]. The studies of our group reported a strong in vitro toxicity of EOs from various botanical genera, such as *Cinnamomum*, *Eucalyptus*, and *Monarda*, to infective juveniles (*J2*) and eggs of the root-knot nematode *Meloidogyne incognita* Kofoid et White (Chitw.), as well as to infective specimens of other phytoparasitic species such as *Pratylenchus vulnus* Allen and Jensen and *Xiphinema index* Thorne and Allen [8,9,10]. Moreover, soil treatments with the same EOs, applied either by fumigation or by irrigation, were highly effective at suppressing *M. incognita* infestation on tomato plants [8,9,10,11].

Lavandin (*Lavandula* × *intermedia* Emeric ex Loisel., syn. *L. hybrida* Reverchon ex Briq) is a natural hybrid of true lavender (*Lavandula angustifolia* Miller, syn. *L. officinalis* Chaix and *L. vera* DC) and the broad-leaved lavender (*L. latifolia* (L. *f*.) syn. *L. spica* Auct.) naturally occurring in the Mediterranean basin and cultivated for its EOs, and it is widely used in perfumery, cosmetics, food processing, and aromatherapy [12,13]. Lavandin EOs obtained from the plant-dried flowering tops are characterized by the presence of terpenoids, such as linalool and linalyl acetate [14], which are primarily responsible for their flavor and numerous biological activities, including antioxidant properties [15] and antimicrobial effects against medical and foodborne pathogens [16,17,18]. However, the content, composition, and consequently biological activities of lavandin EO were found to be highly dependent on the plant cultivar [19] as well as largely affected by agronomical and technical factors such as harvest time, drying temperature, and distillation time [20,21].

Lavandin EO can be a promising raw material for the formulation of crop pesticides, as proved for the insecticidal activity against crop parasites such as *Drosophila suzukii* Matsumura and *Spodoptera littoralis* Boisduval [22,23] or the stored product parasites *Acanthoscelides obtectus* Say and *Sitophilus zeamais* Motschulsky [24,25]. Moreover, EOs or extraction waste materials from lavandin were also reported for contact, repellency, and ovicidal effects on *Tetranychus urticae* Koch (Acari: Tetranychidae) [26], as well as for their toxicity to fungal crop pathogens such as *Alternaria alternata* (Fries) Keissler and *Verticillium dahliae* (Cooke) Wint. [27,28].

The activity of lavandin EOs was poorly and contrastingly documented on phytoparasitic nematodes, as no effect of the whole lavandin EO was found on the root-knot nematode *M. javanica* Treub [22] and the pinewood nematode *Bursaphelenchus xylophylus* Nickle [29,30]. Adversely, a strong activity on *M. javanica* was documented both in vitro and in soil for hydrolate by-products resulting from lavandin EO distillation [31]. The aim of this study was to comparatively evaluate the compositional profile and the in vitro activity on the phytoparasitic nematodes *M. incognita* Kofoid et White and *P. vulnus* of the EOs from three different lavandin cultivars, as well as to assess their in vivo effects in soil infested by *M. incognita* on potted tomato.

## 2. Results

### 2.1. Chemical Constituents of Lavandin EOs

The twenty constituents identified by GC-FID and GC-MS averaged 96.2%, 96.5%, and 93.6% of the total composition in *cv* Abrialis, Rinaldi Cerioni, and Sumiens, respectively (Table 1).

Linalool was the major component of all the three EOs, as accounting for 65.82%, 47.99%, and 40.31% in the *cv* Rinaldi Cerioni, Sumiens, and Abrialis, respectively. Linalool acetate was the second abundant compound in the EO from the *cv* Abrialis (18.35%) and Sumiens (14.86%), while the EO from the *cv* Rinaldi Cerioni was characterized by a significant amount of camphor (11.46% vs. 9.38% and 6.83% in the *cv* Abrialis and Sumiens, respectively). In contrast to the other two cultivars, the EO from the *cv* Sumiens contained a good amount of 1,8-cineole (12.12% vs. 9.95% and 6.96% in the *cv* Rinaldi Cerioni and Sumiens, respectively). Overall, the EO of the *cv* Rinaldi Cerioni was characterized by 93% of oxygenated terpene components, while the other two lavandin cultivars were constituted by lower amounts of oxygenated terpene components (60% and 72% in the *cv* Abrialis and Sumiens, respectively), which are present together with significant amounts of monoterpene esters (20% and 15% in the *cv* Abrialis and Sumiens, respectively).

### 2.2. In Vitro Toxicity Bioassays

A significant mortality of *M. incognita J2* occurred after a 4 h treatment with a 12.5 µg·mL^−1^ concentration of all three EOs, increasing by exposure time and EO concentrations and peaking 82.0%, 95.8% and 89.8% after a 24 h *J2* immersion in 100 µg·mL^−1^ solutions of *cv* Abrialis, Rinaldi Cerioni, and Sumiens EOs, respectively (Table 2). At all the tested concentrations and exposure times, the *cv* Rinaldi Cerioni EO resulted in largely the most toxic to root-knot nematode *J2*, which was followed by *cv* Sumiens EO. These results were also confirmed by values of LC50, as at the 4 h exposure, a 24 µg·mL^−1^ concentration of the *cv* Rinaldi Cerioni EO was enough to kill 50% of *M. incognita J2* vs. the 61.9 and 142.8 µg·mL^−1^ concentrations needed for *cv* Sumiens and Abrialis EOs, respectively. The toxicity of the three EOs was significantly higher compared to that of the chemical control Oxamyl only after a 4 h exposure time, while at the 8 h treatment, this difference persisted only for the 25–100 µg·mL^−1^ concentrations of *cv* Rinaldi Cerioni EO and was annulled at 24 h exposures.

The infective specimens of *P. vulnus* were largely more sensitive to the three lavandin EOs than *M. incognita J2*, as their mortality rates ranged 43.7–65.5%, 58.5–67.7%, and 57.9–75.7% after a 4 h exposure to the EOs from *cv* Abrialis, Rinaldi Cerioni, and Sumiens, respectively (Table 2). The higher toxicity to *P. vulnus* of the three EOs was confirmed by their 4 h LC50 values, resulting in 23.5, 3.1, and 6.1 µg·mL^−1^ vs. 142.8, 24, and 61.9 µg·mL^−1^ of *M. incognita J2*, for *cv* Abrialis, Rinaldi Cerioni, and Sumiens, respectively. The EOs of *cv* Abrialis and Sumiens were consistently more toxic to *P. vulnus* specimens than to *M. incognita J2*, as their mortality rates ranged 43.7–65.5%, 58.5–67.7 and 57.9–75.7%, respectively, after a 4 h exposure (Table 2). Adversely, *P. vulnus* sensitivity to the *cv* Rinaldi Cerioni EO, as well as to the Oxamyl solution, was higher than that to *M. incognita* only at the shortest exposure time.

### 2.3. Egg Hatchability Assay

**Figure 1:**
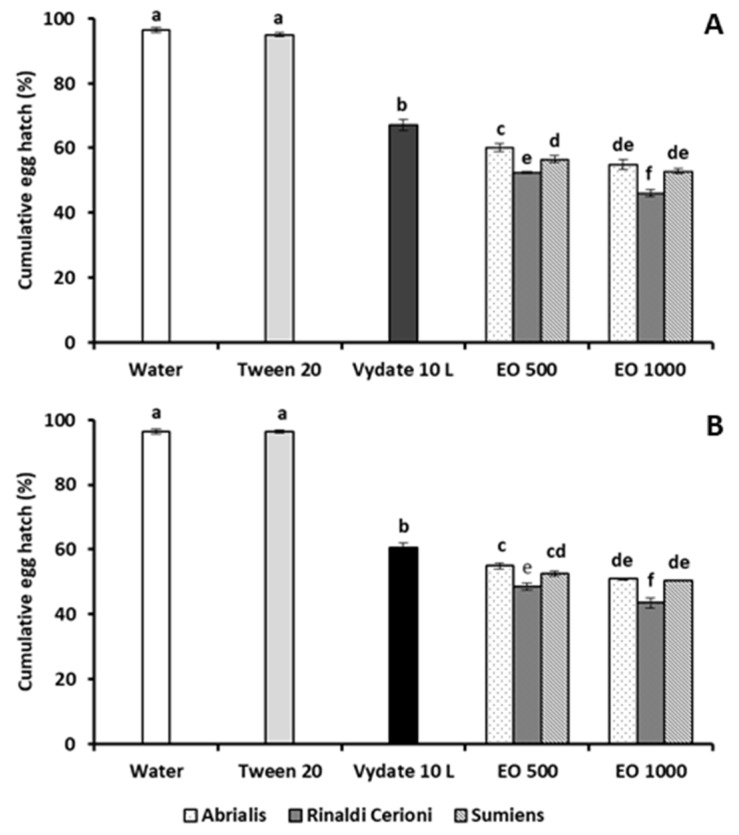
Percentage hatchability (means of four replicates ± SE) of *M. incognita* eggs after 24 (**A**) or 48 h (**B**) exposure of egg masses to 500 (EO 500) and 1000 (EO 1000) μg·mL^−1^ solutions of EOs from lavandin *cv* Abrialis, Rinaldi Cerioni, and Sumiens. Bars marked with the same letters are not significantly different according to Least Significant Difference Test.

The hatchability of *M. incognita* eggs was always significantly reduced by treatments with the three lavandin EOs, without any statistical difference between the 24 and 48 h exposure (Figure 1).

The lowest egg percentage hatch was recorded after the egg mass immersion in a 100 µg·mL^−1^ solution of the *cv* Rinaldi Cerioni EO, 46.1% and 43.6% vs. 96.3% and 96.4% of water control, respectively.

### 2.4. Experiment in Soil

Soil treatments with the three lavandin EOs strongly reduced the density of *M. incognita* eggs and *J2* both on tomato roots and in soil, according to a dose–effect relationship (Table 3). Moreover, the 100 µg·kg^−1^ soil dose of the three EOs was also significantly more suppressive on *M. incognita* than the treatment with Oxamyl. All EO treatments but the 62.5 µg·kg^−1^ dose of *cv* Abrialis EO also caused a significant reduction of gall formation on tomato roots, although with less clear differences among the EO doses.

Compared to the non-treated control, the growth of tomato plant aerial parts after soil treatments with the 62.5–250 µg·kg^−1^ soil doses of the three lavandin EOs also resulted in a significant increase of tomato green biomass, whereas no statistical difference was generally found at the two highest treatment doses (Table 3). Adversely, the growth effects on a plant root system were always statistically significant except for the 1000 µg·kg^−1^ soil dose of *cv* Abrialis and Sumiens EO.

## 3. Discussion

The tested lavandin EOs showed a strong activity against both the root-knot nematode *M. incognita* and the root lesion parasite *P. vulnus*. Contrastingly, the few previous literature reports described a low nematicidal effect of lavandin EOs. In particular, a commercial EO from lavandin *cv* Super was found poorly active on the root-knot species *M. javanica*, although showing a strong activity against the Egyptian cotton leafworm *S. littoralis* [22]. Analogously, a poor toxicity to the pinewood nematode *B. xylophilus* was repeatedly stated for the EO from lavandin *cv* Grosso [29,30]. In contrast, a hydrolate by-product from the vapor–pressure extraction of EO from lavandin *cv* Super showed a strong in vitro nematicidal activity against *M. javanica* both in terms of *J2* mortality and egg-hatching suppression, and it also significantly reduced the infection and reproduction of *M. javanica* population on tomato when applied to soil [31].

According to the results from this study, nematicidal effects of lavandin EOs can largely vary among the source varieties as well as among the nematode species. The EO from *cv* Rinaldi Cerioni was significantly more toxic than that from *cv* Abrialis and Sumiens EOs to both *M. incognita* and *P. vulnus* in the in vitro assays, while differences among the three cultivars were less evident when the EOs were applied to soil infested by *M. incognita*.

This is the first report of a nematicidal activity of EOs from these three lavandin cultivars, as they were comparatively tested only for their antimicrobial activity against foodborne pathogens such as *Listeria monocytogenes* L. and *Salmonella enterica* Kauffmann & Edwards [17].

**Figure 2:**
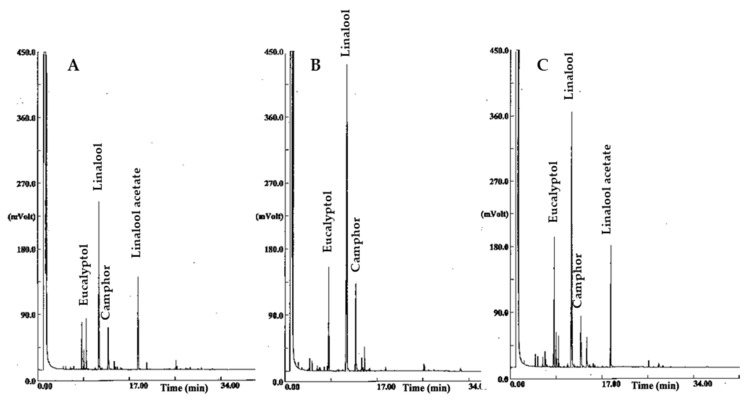
Gas chromatograms of EOs from lavandin *cv* Abrialis (**A**), Rinaldi Cerioni (**B**), and Sumiens (**C**). Major components are indicated.

Plant EOs are a complex mixture of lipophilic molecules highly functionalized to produce a variety of chemical structures such as alcohols, aldehydes, ketones, esters, and other chemical types that specifically characterize some plants families and firstly contribute to their EO flavor. Nevertheless, due to their chemical diversity, plant EOs display several biological and pharmacological activities [32,33]. Many studies also from our group have demonstrated that the toxic effects of EOs against nematodes are influenced by their chemical profile [2,6,8,9,10,34,35,36]. *Lavandula* hybrids are highly aromatic plants, and lavandin EO is generally made up by linalyl acetate (19–26%), linalool (20–23%), 1,8-cineole (10%), and camphor (12%), as the main components [37]. The relative content of these compounds identifies the different cultivars of lavandin EO. Consistently, lavandin EOs investigated in our study are distinguished by different amounts of linalyl acetate, linalool, 1,8-cineole, and camphor (Figure 2), according to the same average composition detected in a previous study on EOs from the same source plant material [17].

Comparative analyses of chemical composition of plant EOs and their nematicidal activity allowed outlining some structure–activity relationships mainly depending on the chemical features of the dominant components [2]. Thus, it has been documented that the toxic effects against nematodes are influenced by the type and functional groups in the molecule, and the nematicidal activity is generally enhanced by the oxygenation and unsaturation of the molecule. The highest nematotoxic activity disclosed by the EO from the *cv* Rinaldi Ceroni compared to the other two tested EOs should then reasonably be ascribed to the very high presence of oxygen-containing molecules (93%) compared to the *cv* Abrialis and Sumiens EOs, which instead contain fewer oxygenated molecules but higher quantities of less reactive esterified constituents (20% and 15% in the *cv* Abrialis and Sumiens EO, respectively). Thus, as already documented for EOs from other aromatic species, the synergistic action of the main oxygenated constituents, linalool, camphor, and 1,8-cineole should be taken as responsible for the highest nematotoxic effect of the EO from the lavandin *cv* Rinaldi Ceroni. Overall, the good nematicidal activity of the three varieties of lavandin oil used in this study should also be related to the acyclic structure of linalool, the major component, as demonstrated for other EOs containing similar chemical structures such as for example citronellol and/or geraniol [2,8].

Differences in sensitivity to EOs and their components among the phytonematode species were already observed in previous studies of our group [9,10] as well as of other authors [38] and can be mainly attributed to a different anatomy and feeding behavior [39,40]. In particular, *P. vulnus* was found to be less sensitive than *M. incognita* and *X. index* to the EOs from *A. herba-alba*, *R. officinalis*, and *T. saturejoides* [10] as well as to EOs from *Monarda* species [9], whereas the *C. sinensis* EO was poorly active on *M. incognita* and more toxic to *P. vulnus* [10].

Although many EOs compounds were found to be toxic or repellent to phytoparasitic nematodes, the available structure–activity studies still provide very few insights into the mode of action of these compounds. Therefore, hypotheses suggested for the mechanisms of EOs components’ nematicidal activity were generally based on pharmacological effects observed in insects [6]. Several EOs monoterpenes were demonstrated as competitive inhibitors of acetylcholinesterase isolated from different insect species [41,42], while other studies also documented a functional disruption of detoxifying systems in insects by EOs’ compounds such as thymol, eugenol, pulegone, terpineol, and citronellal [43,44]. The octopaminergic system, as playing a key role as a neurotransmitter, neurohormone, and neuromodulator in invertebrate systems, was also indicated as a target of the action of EOs constituents in insects [45,46]. The change of permeability of cell membranes by the low-molecular weight and highly lipophilic EOs components, as easily passing through and causing disruption to the cell organization, was also recognized as a further mechanism of EOs activity both on insects and fungi [47,48].

Suppression of the nematode infestation in soil treated with the three lavandin EOs is in full agreement with the strong suppressive effects on *M. incognita* gall formation and egg multiplication on tomato roots constantly observed after soil treatments with a wide range of EOs [2]. However, a field exploitation of the strong nematode suppressiveness of EOs needs the development of agrochemical formulations, ensuring their long-term stability and biological activity. This issue has been widely investigated on lavandin EOs, as tested for encapsulation with biopolymers by supercritical fluids [49], liposome incorporation [50], or emulsification with modified starches [51].

## 4. Materials and Methods

### 4.1. Essential Oils

Plants of lavandin cultivars Abrialis, Rinaldi Ceroni, and Sumiens were cultivated at the Herb Garden “Augusto Rinaldi-Ceroni” (Casola Valsenio, Ravenna, Emilia-Romagna region, Italy). Voucher specimens of each cultivar are deposited at the Department of Agricultural and Food Sciences of University of Bologna (Bologna, Italy), with identification numbers B130908, B130909, and B30910, respectively. EOs were extracted from alfalfa mosaic virus (AMV)-free lavandin plants to avoid changes during the flowering period [52].

Plant fresh leaf and flower material was collected in the second week of August, and EOs were immediately extracted by a 2 h steam distillation with a commercial Clevenger apparatus (Albrigi, Verona, Italy). EO yields were 1.67%, 1.25%, and 1.50% of fresh weight for *cv* Abrialis, Rinaldi Ceroni, and Sumiens, respectively. EOs were dried overnight over anhydrous Na_2_SO_4_ and then kept in the refrigerator until ready to be analyzed.

### 4.2. Chemical Analysis of Essential Oils

EOs from the three lavandin cultivars were analyzed with a Trace GC-FID Ultra Thermo Finnigan gas chromatograph equipped with an Agilent DB-5 (J & W Scientific, Milan, Italy) fused silica capillary column (30 m × 0.25 mm; 0.25 µm film thickness). Adopted analytical conditions were as follows: detector temperature 300 °C; the column temperature was programmed from 60 °C (5 min isothermal) to 280 °C (30 min isothermal) at 4 °C/min. Hydrogen was the carrier gas (35 kP; 2.0 mL/min). Data were processed using a Chrom-Card 32-bit version 2.0 computing software. Analyses were run in the cold on-column mode. Quantitative composition was expressed as a percentage value for each of the EOs constituents from the total peak area detected by GC-FID analyses without using correction factors.

GC-MS analyses were carried out with a Hewlett Packard 6890 (MSD)-5973 (GC) GC-MS System interfaced with a HP Chemstation (Agilent. Scientific Instruments, Milan, Italy) The following analytical parameters were used: column oven program 60 °C (5 min isothermal) to 240 °C (15 min isothermal) at 3 °C min^−1^; injector, 280 °C. Helium was the carrier gas (flow rate, 1 mL·min^−1^). Chromatographic separation was performed with a HP-5 MS capillary column (30 m × 0.25 mm; 0.25 µm film thickness); MS operating conditions were as follows: ion source, 70 eV; ion source temperature, 200 °C; mass spectra acquisition, over 40–800 amu range at 1 scan·s^−1^. The ion source was operating in the electron impact mode. Samples (1 µL) were injected using the splitless sampling technique.

Identification of the constituents of each EO was based on comparison with GC retention times of authentic reference compounds in combination with arithmetic indexes (AI) and by means of reference mass spectra from standard compounds and/or from NIST (National Institute of Standards and Technology) mass spectral library files [53,54]. Arithmetic Indexes (Table 1) were calculated (*AI calc*) in reference to n-alkanes (C_6_–C_32_) under the same GC conditions as those for the EOs and compared with published AI (*AI tab*) [53].

### 4.3. Nematode Populations

The population of *M. incognita* was previously reared on tomato *cv* Regina di Fasano in a glasshouse maintained at 25 ± 2 °C. Mature egg masses were handpicked from the infested tomato roots and incubated in distilled water in a growth chamber at 25 °C. Emerged juveniles were collected and stored at 5 °C until used.

The population of *P. vulnus* was recovered from olive roots at Valenzano (province of Bari, Apulia region) and then reared on carrot disks [55]. Mixed-age infective specimens of *P. vulnus* were recovered from carrot disks by washing with sterile distilled water and used immediately.

### 4.4. In Vitro Toxicity Assay

A 0.5 mL volume of a nematode suspension in distilled water, containing about 100 individuals of *M. incognita J2* or *P. vulnus* mixed stages, was pipetted in 1.5 mL Eppendorf tubes. Another 0.5 mL volume of 25, 50, 100, and 200 μg·mL^−1^ EOs’ solutions in 0.3% Tween 20 distilled water was added to each Eppendorf tube containing the nematode suspensions to obtain 12.5, 25, 50, and 100 μg·mL^−1^ final test concentrations. Nematodes were exposed to each EO concentration for 4, 8, or 24 h. Four replicates were provided for each concentration at each exposure time. Distilled water, a 2 mL·L^−1^ water solution of the nematicide Oxamyl (10% a.i.), and 0.3% Tween 20 were included as controls.

At the end of each exposure period, nematodes were observed under a light microscope, checking the number of motile and paralyzed specimens, and then transferred to distilled water for further 72 h. The permanence of immobility after the immersion in water, as stated by a new microscopical observation, was assumed as a confirmation of nematode mortality. Nematode percentage mortality was calculated by Abbott’s formula *m* = 100 × (1 − *nt*/*nc*), in which *m*, percent mortality; *nt*, number of viable nematodes after the treatment; *nc*, number of viable nematodes in the water control [56].

### 4.5. Egg Hatchability Test

Egg masses from the same population of *M. incognita* used in the toxicity assays were picked from the infested tomato roots and put in 1.5 mL Eppendorf tubes. Each tube contained 30 egg masses (420 eggs per mass), immersed in 0.5 mL of distilled water. A 0.5 mL volume of a 0.3% Tween 20 water solution containing 1000 or 2000 μg·mL^−1^ concentrations of the three lavandin EOs was added to each Eppendorf tube as to reach final 500 and 1000 μg·mL^−1^ test concentrations. As in the previous experiment, distilled water, the 2 mL·L^−1^ water solution of Oxamyl, and 0.3% Tween 20 were used as controls. Four replicates were provided both for EO treatments and controls.

After a 24 or 48 h exposure to each EO solution, egg masses were rinsed in distilled water, placed in 2 cm diameter sieves (215 μm aperture size) arranged in 3.5 cm diameter Petri dishes, and submerged with 3 mL of distilled water. Then, a hatching test was carried out in a growth chamber at 25 °C for five weeks. At weekly intervals, the emerged *J2* were removed and microscopically counted, while egg masses were repeatedly washed with sterile water, checked for the presence of microbial contaminations, and covered with fresh distilled water. At the end of the five-week hatching period, egg masses were removed from each sieve and dissolved by a 3 min shaking in a 1% sodium hypochlorite aqueous solution [57] so as to count the unhatched eggs under an optical microscope. Egg hatchability was expressed as cumulative percentages of *J2* emerged during the hatching test on total eggs forming the egg masses.

### 4.6. Experiment in Soil

A sandy soil (64.4% sand, 18.7% silt, 16.9% clay, 0.8% organic matter, 7.5 pH) was steam sterilized and then mixed with finely chopped tomato roots infested by the same *M. incognita* population used in the in vitro bioassays to reach a 20 eggs and *J2* mL^−1^ soil initial population density. The infested soil was placed into 1.5 L clay pots and then treated with 62.5, 125, 250, 500, or 1000 μg·kg^−1^ soil rates of the EOs from the three lavandin cultivars suspended in a 400 mL volume of 0.3% Tween 20 water solution. Controls were represented by non-treated soil, either infested by *M. incognita* or non-infested, and by soil treated with a 2 mL·kg^−1^ soil rate of the same liquid formulation of Oxamyl used in the in vitro experiments, applied three days before transplanting. Pots were arranged in a randomized block design, with five replicates of each treatment and controls, on the benches of a greenhouse maintained at a 25 ± 2 °C constant temperature.

A 1-month-old seedling of tomato *cv* Regina di Fasano was transplanted in each pot three weeks after the treatments with lavandin EOs. Plants were maintained in the greenhouse for two months, after which they were uprooted, and the fresh weight of aerial parts and roots of each plant was recorded. Gall formation due to *M. incognita* infestation was evaluated on each tomato root according to the 0–5 Taylor and Sasser’s scale [58], in which 0 = no galls, 1 = 1–2 galls, 2 = 3–10 galls, 3 = 11–30 galls, 4 = 31–100 galls, and 5 > 100 galls. The final population density of *M. incognita* in each pot was determined by processing each tomato root with a 1% aqueous solution of sodium hypochlorite [57] and extracting nematodes from a 500 mL soil sample from each pot by Coolen’s method [59] and then microscopically counting eggs and *J2*.

### 4.7. Statistical Analysis

All the experiments were repeated twice, and the data from the two experimental runs were pooled, due to no presence of significant experiment x treatment interactions [56]. Pooled data were subjected to one or two-way ANOVA, and means were compared by Fisher’s Least Significant Difference Test at *p* ≤ 0.05, using the software program PlotIT 3.2 (Scientific Programming Enterprises, Haslett, MI, USA). The LD_50_ of the three EOs on *M. incognita J2* was calculated by the probit analysis [56] of mortality data from the in vitro bioassay.

## 5. Conclusions

Data from this study indicated that lavandin EOs, as most of the EOs tested in previous studies of our research group and other authors, can exert a strong toxicity to phytoparasitic nematodes even at very low concentrations, and therefore, they can be a further source of new nematicidal formulations alternative to synthetic nematicides. A significant role in this kind of industrial exploitation of lavandin cultivation can be played by its easy adaptability to various pedoclimatic conditions, the large EO production and, finally, the added value provided by side products of EO extraction, such as hydrolates. Moreover, the recent arrival on the market of new nematicides based on synthetic homologues of EOs terpene components indicates that lavandin EOs could be exploited also as a model for new nematicidal mixtures of synthetic derivatives of their terpene constituents.

The technical and economical optimization of lavandin EO production is strictly related to the choice of suitable lavandin varieties, due to the intervarietal variation of EOs nematicidal activity also emerged in this study. More specifically referring to the varieties tested in our experiments, the influence of viral diseases on lavandin secondary metabolism can be considered an important factor for the standardization of OE production, since it may affect its pharmacological or functional properties. It is known that the most common viral disease affecting the genus *Lavandula* in Europe is due to alfalfa mosaic virus (AMV), causing yield decreases ranging from 46.8% to 4.6%, with a 41% peak loss in *cv* Abrialis [52]. Moreover, the nematotoxic activity of EOs from virus-infected plants could be compromised also by decrease of oxygenated terpenes, ranging from 43.9% to 39.2% and 33.47% to 30.36% for linalool in lavandin *cv* Sumiens and Abrialis, respectively [52].

Another point in favor of addressing lavandin crops to the production of new EO-based nematicides can be represented by the availability of an extended literature on technical formulations more suitable for a controlled release and a slow degradation of EOs active compounds in soil, as prolonging and enhancing the nematicidal effect of soil treatments with EO formulations.

## Figures and Tables

**Table 1:** Chemical composition of EOs from lavandin *cv* Abrialis, Rinaldi Cerioni, and Sumiens as determined by GC-FID and GC-MS analyses.

Compound *	*AI calc* **	*AI tab* **	Mean Percentages ± SD (*n* = 3)		
			Abrialis	Rinaldi Cerioni	Sumiens
α-Pinene	931	932	0.28 ± 0.006	0.69 ± 0.003	0.70 ± 0.002
Camphene	943	946	0.33 ± 0.01	0.62 ± 0.006	0.63 ± 0.002
β-Pinene	974	974	0.29 ± 0.005	0.34 ± 0.002	0.51 ± 0.29
Myrcene	989	988	0.35 ± 0.01	0.21 ± 0.004	-
Limonene	1023	1024	0.52 ± 0.01	0.36 ± 0.008	0.69 ± 0.01
1,8-cineole	1025	1026	6.96 ± 0.15	9.95 ± 0.12	12.12 ± 0.05
β-(*Z*)-Ocimene	1033	1032	2.99 ± 0.04	-	3.00 ± 0.02
β-(*E*)-Ocimene	1044	1044	8.34 ± 0.11	-	0.43 ± 0.01
Terpinolene	1087	1086	0.43 ± 0.004	0.30 ± 0.01	0.30 ± 0.01
Linalool	1095	1095	40.31 ± 0.99	65.82 ± 0.12	47.99 ± 0.07
1-Octen-3-ol-acetate	1111	1110	0.32 ± 0.004	-	-
Camphor	1143	1141	9.38 ± 0.25	11.46 ± 0.04	6.83 ± 0.02
Borneol	1166	1165	1.71 ± 0.09	1.76 ± 0.02	4.31 ± 0.03
Lavandulol	1164	1165	0.77 ± 0.01	0.57 ± 0.02	-
Terpinen-4-ol	1174	1174	0.62 ± 0.02	2.86 ± 0.01	0.30 ± 0.003
α-Terpineol	1187	1186	0.50 ± 0.01	0.21 ± 0.01	0.45 ± 0.01
Linalool acetate	1255	1254	18.35 ± 1.02	0.46 ± 0.16	14.86 ± 0.17
Lavandulyl acetate	1290	1288	1.40 ± 0.02	-	-
β-Caryophyllene	1418	1417	1.80± 0.09	0.70 ± 0.06	0.50 ± 0.06
β-Ylangene	1421	1419	0.60± 0.08	0.18 ± 0.09	-
Total yield			96.25	96.49	93.62

* Compounds are listed according to their elution time. ** *AI calc* = arithmetic indexes calculated (*AI calc*) in reference to *n*-alkanes (C_6_–C_32_) under the same GC conditions as that for the EOs; *AI tab* = published arithmetic indexes.

**Table 2:** Percentage mortality (means of four replicates ± SE) of *Meloidogyne incognita J2* and mixed infective stages of *Pratylenchus vulnus* after 4, 8, or 24 h exposure to a 12.5–100 μg·mL^−1^ range of concentrations of EOs from lavandin *cv* Abrialis, Rinaldi Cerioni, and Sumiens.

Concentration (µg·mL^−1^)	4 h		8 h		24 h	
	*M. incognita*	*P. vulnus*	*M. incognita*	*P. vulnus*	*M. incognita*	*P. vulnus*
	Abrialis					
12.5	15.2 ± 0.9	43.7 ± 1.6	26.2 ± 0.3	50.4 ± 2.1	36.7 ± 2.7	56.8 ± 4.5
25	23.3 ± 2.0	50.9 ± 1.6	35.9 ± 1.3	61.2 ± 1.4	40.8 ± 1.4	65.9 ± 1.2
50	34.3 ± 2.2	62.2 ± 3.2	40.6 ± 1.3	67.0 ± 2.1	72.4 ± 2.4	70.9 ± 2.3
100	43.3 ± 1.9	65.5 ± 3.1	71.5 ± 0.9	73.0 ± 1.5	82.0 ± 1.5	78.8 ± 0.8
LC50	142.8	23.5	48.6	11.8	24.9	7.4
	Rinaldi Cerioni					
12.5	34.3 ± 1.5	58.5 ± 4.9	49.0 ± 1.2	64.3 ± 0.4	81.7 ± 0.5	67.6 ± 1.8
25	46.1 ± 0.3	59.1 ± 1.9	72.6 ± 1.6	66.2 ± 1.8	89.5 ± 1.6	73.7 ± 1.6
50	73.3 ± 1.7	59.2 ± 2.2	84.6 ± 1.7	75.7 ± 16.1	93.9 ± 2.6	81.9 ± 4.4
100	82.6 ± 1.5	67.7 ± 3.6	95.1 ± 0.5	77.9 ± 1.7	95.8 ± 1.9	86.1 ± 0.8
LC50	24.0	3.1	12.6	2.9	1.2	3.1
	Sumiens					
12.5	15.9 ± 0.5	57.9 ± 2.8	32.5 ± 2.9	70.5 ± 1.4	40.9 ± 3.6	74.6 ± 0.9
25	26.6 ± 2.7	64.0 ± 1.2	41.4 ± 1.1	72.1 ± 3.7	60.6 ± 2.5	77.0 ± 2.1
50	44.0 ± 2.7	66.1 ± 2.4	56.6 ± 1.3	75.3 ± 0.6	76.3 ± 2.3	81.1 ± 1.9
100	63.0 ± 1.3	75.7 ± 3.1	78.2 ± 0.7	83.1 ± 1.0	89.8 ± 2.8	85.7 ± 0.6
LC50	61.9	6.1	31.5	1.1	17.4	0.5
Oxamyl *	9.9 ± 0.5	34.9 ± 0.5	49.4 ± 1.2	59.4 ± 1.2	91.1 ± 1.5	71.1 ± 1.4
Tween 20 **	0.0 ± 0.0	1.1 ± 0.5	0.9 ± 0.6	1.2 ± 0.1	1.3 ± 1.0	1.5 ± 0.3
Water	0.0 ± 0.0	1.1 ± 0.8	0.3 ± 0.2	1.3 ± 0.7	0.4 ± 0.4	2.1 ± 1.4
LSD (*P* = 0.05)	4.5	7.2	3.7	12.7	5.9	6.0

* 2 mL·L^−1^; ** 0.3%.

**Table 3:** Effects (means of five replicates ± SE) of soil treatments with 62.5–1000 µg·kg^−1^ soil rates of EOs from lavandin *cv* Abrialis, Rinaldi Cerioni, and Sumiens on *Meloidogyne incognita* infestation on tomato *cv* Regina di Fasano of the root-knot nematode and tomato plant growth.

EO Dose (µg·kg^−1^ Soil)	Nematode Population (Eggs and *J2*)		Root Gall Index (0–5)	Plant Fresh Weight (g)	
	g^−1^ Roots	mL^−1^ Soil		Aerial Parts	Roots
	Abrialis				
62.5	157.0 ± 6.8	10.3 ± 0.2	4.5 ± 0.3	38.9 ± 3.5	16.2 ± 2.0
125	117.6 ± 0.7	8.3 ± 0.3	4.0 ± 0.0	45.2 ± 6.9	15.9 ± 2.0
250	94.1 ± 1.1	6.2 ± 0.2	3.5 ± 0.3	35.9 ± 5.5	16.7 ± 2.1
500	72.1 ± 0.4	5.0 ± 0.2	3.5 ± 0.3	31.5 ± 2.7	13.9 ± 1.7
1000	54.1 ± 2.1	3.8 ± 0.1	3.0 ± 0.0	31.0 ± 2.7	11.9 ± 1.5
	Rinaldi Cerioni				
62.5	124.1 ± 3.1	8.3 ± 0.1	4.2 ± 0.2	41.1 ± 3.4	20.6 ± 2.6
125	96.9 ± 1.2	6.7 ± 0.2	3.7 ± 0.2	47.6 ± 4.2	20.2 ± 2.5
250	76.0 ± 2.4	4.9 ± 0.2	3.2 ± 0.2	40.5 ± 3.3	17.5 ± 2.2
500	64.4 ± 2.3	4.2 ± 0.1	3.2 ± 0.2	30.0 ± 2.6	15.6 ± 1.9
1000	37.6 ± 1.3	2.6 ± 0.1	2.5 ± 0.3	28.5 ± 2.5	13.7 ± 1.7
	Sumiens				
62.5	141.2 ± 0.6	9.2 ± 0.4	4.2 ± 0.3	40.0 ± 3.2	18.0 ± 2.2
125	102.6 ± 1.6	7.1 ± 0.2	3.7 ± 0.2	46.8 ± 4.1	18.2 ± 1.5
250	82.9 ± 1.3	5.3 ± 0.2	3.2 ± 0.3	38.3 ± 3.0	17.0 ± 2.1
500	70.0 ± 0.6	4.8 ± 0.1	3.2 ± 0.2	30.9 ± 1.4	14.4 ± 1.8
1000	44.5 ± 1.4	3.1 ± 0.1	2.7 ± 0.2	29.4 ± 2.6	12.8 ± 1.6
Oxamyl (2 mL·kg^−1^ soil)	61.2 ± 1.5	4.8 ± 0.2	3.0 ± 0.0	29.0 ± 1.8	11.9 ± 1.5
Tween 20 (0.3%)	205.8 ± 4.0	15.6 ± 0.8	5.0 ± 0.0	23.4 ± 1.4	7.4 ± 1.0
Non-treated	206.8 ± 2.3	15.9 ± 0.2	5.0 ± 0.0	21.7 ± 0.7	7.6 ± 0.7
Non-infested	-	-	-	41.2 ± 2.5	18.1 ± 1.4
LSD (*p* = 0.05)	6.9	0.7	0.6	9.5	5.2

## Data Availability

The data presented in this study are available on request from the corresponding author. The data are not publicly available due to privacy restrictions.

## Sample Availability

Samples of the compounds are available from the authors.
